# Fall into science: increasing opportunities to enter the physician-scientist pathway

**DOI:** 10.1172/JCI176035

**Published:** 2024-01-02

**Authors:** Yentli E. Soto Albrecht

**Affiliations:** Perelman School of Medicine at the University of Pennsylvania, Philadelphia, Pennsylvania, USA.

“How did you fall into science?”

It is a simple enough question, with a usually straightforward answer. What drew you in, and why did you stay? But the question makes a series of assumptions — that the attraction to this calling serves as a gravitational pull and that this pull is sufficient to drive you home.

The American Physician Scientists Association (APSA) is the United States’ premier organization dedicated to supporting physician-scientists in training, led by trainees, for trainees. We have over 1,800 members, ranging from undergraduate associate members to current trainees, residents, and fellows. It was my privilege to serve as APSA president from 2022 to 2023.

“How did you fall into science?” is a question I’ve been asking fellow scientists lately. Seated around the table at an international conference last fall, I listened as leaders in their field, in turn, gave their answers. Despite differences in cultures and trajectories, their stories shared a common thread: an opportunity offered, a foot in the door — the start of a new chapter. I will explore here the concept of the “foot in the door” toward physician-scientist training and relate it to my own experience. I will expand upon APSA’s efforts to diversify the physician-scientist workforce by creating opportunities at a critical entry point into this pathway.

I reflect on my journey toward the calling of becoming a physician-scientist. I was a sophomore in college when I discovered the MD-PhD trajectory from a fellow student. By then, I had cold-emailed professors and doggedly attended office hours for a year trying to join a research lab. I knew I wanted to develop the skill set to think of my own hypotheses and design and accomplish experiments that would give me answers. I was feeling exasperated. How could I enter the field of molecular biology if I couldn’t land a research experience? My mother’s experience at a university in Puerto Rico weighed on me, where she switched majors because her mentors convinced her that biology wasn’t a subject where she could excel. Was this to be my fate too?

In the spring of 2014, I became a nominee for a prestigious undergraduate award — in hindsight, my foot in the door. I leveraged this nomination to obtain a research experience in the lab of Bonnie Bassler, the pioneer of bacterial quorum sensing. I felt privileged by the opportunity to marry my curiosity with the tools to answer my questions. Most importantly, this was the gateway to three substantial research experiences and my success in applying to MD-PhD programs four years later. I am grateful for the opportunities I had but also deeply aware that chance exposed me to and set me on this pathway.

Luckily, APSA doesn’t rely on chance. Our initial approach to equality in this regard was informed by justice, equity, diversity, and inclusion (JEDI) efforts. Our logic: If we only support physician-scientist trainees arising from opportunities afforded through family and connections, our ability to capture a diverse trainee population is severely limited. And this is indeed what we see.

To this day, MD-PhD matriculants are most likely from families with an annual household income of over $100,000. They are most likely to have highly educated parents relative to the United States population, many with at least one doctorate (1). MD-PhD applicants and alumni are most likely to be men and either White or Asian compared with the combined numbers of ethnicities and races underrepresented in science and medicine (URM). This long-standing problem has only mildly improved over decades (2, 3). These studies do not consider other pathways to becoming a physician-scientist, including DO-PhD and DO-MD single-degree physician-scientists, where representation tends to be lower. Mora and colleagues examined the time it would take to reach a scenario in which physician diversity matches that of the US population. They postulated that, even in the unlikely scenarios in which Black and Latinx/Hispanic matriculation into MD programs quadruples, triples, or doubles, it would take between 25 and 90 years to match existing population diversity (4).

Clearly, doing nothing is not an option — and APSA has felt this way for years. As a direct result of the 2014 data released by the NIH detailing these gender, racial, ethnic, and socioeconomic disparities in physician-scientist representation (5), APSA formed the physician-scientist trainee diversity working group (PST-DWG). This working group included representatives from partnering organizations and was tasked with guiding our response to this issue. APSA subsequently partnered with the Burroughs Wellcome Fund (BWF) in 2019 for the first Physician-Scientist Trainee Diversity Summit, bringing together 66 stakeholders to answer the question, “How do we develop a robust and diverse physician-scientist workforce?”

A vital aspect of the summit was brainstorming approaches to diversify the physician-scientist workforce. The cumulative ideas covered all aspects of physician-scientist training, from increasing awareness in schoolchildren to improving MD-PhD admissions transparency, training and retention, mentoring and community building, and needs assessment studies to better understand barriers to achieving physician-scientist success at each stage of training (6). This diversity summit also held personal importance, as it was my first introduction to APSA.

I joined these efforts in the summer of 2019 and eventually led this work within our organization: as a member of the diversity ad hoc committee in 2019, as chair of this committee in 2020, as president-elect of APSA in 2021, and as president in 2022. I am incredibly proud of the work we accomplished on all these fronts, grateful for the opportunity to support these efforts and the trainees driving them, and excited for the work we have yet to do. I’ll briefly touch on this work while elaborating on the foot-in-the-door problem and APSA’s ongoing approach to solving it.

What does the foot in the door toward physician-scientist training necessitate? First, awareness that the door exists. This is what we refer to as exposure to the physician-scientist pathway. Then, entering requires opportunity, experience, skill set, and guidance. APSA’s diversification efforts recognize that individuals from URM backgrounds face increased barriers at both entry requirements.

We combat the barriers to exposure through outreach to schoolchildren and undergraduates. To reach schoolchildren nationwide, we created Mini Lessons by Medical Scientists in 2020 (7), a series of YouTube videos by physician-scientist trainees explaining our chosen pathway and scientific topics from the perspective of translational research. These have garnered thousands of views to date. We also created a toolkit for APSA’s more than 70 local chapters that includes specific steps for working with local schools to expose children to this career path, complete with sample programming and funding mechanisms for such diversity initiatives through our partner, BWF. To reach undergraduates and postgraduates in this capacity, we created interactive sessions such as “A Day in the Life of Physician-Scientists” that grow awareness about this career and provide an opportunity for questions and answers from successful physician-scientists themselves. These started in 2015 and continue annually, engaging hundreds of participants, and are available for free on our website for later viewing.

Barriers to entering the physician-scientist “doorway” are a bit more complicated. Knowing that the door exists isn’t enough; if you don’t already have the resources, preparation, and experience to enter, gravity won’t work the same way. Overcoming these barriers requires opportunities, mentors, time, and money. And then, in the spring of 2020, the COVID-19 pandemic magnified existing barriers — research opportunities evaporated, as did many jobs. Applicants from families who could not pivot to a virtual workforce found themselves without the finances to apply to medical school or, in many cases, scrambled to provide for their families. So how does a nonprofit run by trainees tackle this monumental issue?

In April 2020, two people approached me with different ideas. Within 40 days, we established two interventions led by APSA volunteers: the Virtual Summer Research Program (VSRP) and the Applicant Interactive Session series. By pairing mentors (e.g., principal investigators and graduate students) with undergraduates from URM backgrounds, APSA aimed to provide future applicants with critical virtual research experiences and resources needed to pursue a physician-scientist career path. With support from BWF, APSA continued VSRP for second and third iterations in 2021 and 2022, supporting over 200 individuals from URM backgrounds who were interested in this pathway of research experiences (8). Our Applicant Interactive series was built to provide timely support to applicants through virtual question-and-answer sessions. These sessions occur with MD-DO-PhD program directors and current trainee panelists and are synchronous with the MD-DO-PhD application cycle. It has totaled 31 sessions and over 3,500 registered live participants since its inception in 2020, and we continue to receive engagement with the uploaded recording years later (9). In addition to providing research opportunities to hundreds and guidance on the application process to thousands of future and current applicants, we host a robust mentorship program for future physician-scientist trainees.

Our undergraduate mentorship program has nearly doubled since 2018. From 2020 through 2022, we supported a combined number of more than 1,500 mentor-mentee (current trainee–future applicant) pairs. Importantly, over 60% of the mentees were female, 35% were from URM backgrounds, and over 60% came from households with annual incomes below $50,000 (10). Through these programs, we are specifically working to boost underrepresented identities within the physician-scientist workforce. To assess and evaluate the effectiveness of the Undergraduate Mentorship Program, VSRP, and Applicant Interactive Sessions, APSA obtained IRB approval to collect longitudinal participant data. Ultimately, the goal is to assess these programs as an intervention to increase the success of applicants in physician-scientist careers and increase the matriculation of applicants from URM backgrounds. Analysis of early data shows that APSA’s interventions have the potential to directly address our goal of increasing the diversity of applicants in the physician-scientist training workforce.

APSA recognizes that more than simply supporting entry into the pathway is needed. We continue working with our partners to assess barriers to retention and progression that affect the physician-scientist workforce, highlighting those that disproportionately disadvantage certain trainees. Since 2020 alone, APSA has supported more than five needs and intervention assessments. We have published our findings, including “Factors associated with underrepresented minority physician scientist trainee career choices (11),” “The impact of COVID-19 on physician-scientist trainees and faculty in the United States: a national survey (12),” and “The Virtual Summer Research Program: supporting future physician-scientists from underrepresented backgrounds (8).”

Beyond opportunities for community-building at our national and regional meetings, we have hosted several in-person and virtual meetings focused on providing resources to trainees from URM backgrounds in the last two years. These include “Demystifying the Black Physician-Scientist: Success and Perseverance Seminar,” a session in partnership with the American Association of Black Physician Scientists, as well as “Financing Your Future: for Physician-Scientist Trainees” and “First Generation Physician-Scientist Trainee Community Building Event.” This year, we worked closely with our Joint Meeting partners, the American Society for Clinical Investigation (ASCI) and the Association of American Physicians (AAP), to increase mentorship opportunities for current trainees. APSA members had the opportunity to network with early career physician-scientists at the ASCI reception. They had opportunities for further discussion at the APSA mentorship and specialty interest breakfasts. In addition, AAP partnered with APSA for an inaugural 2023 Joint Meeting mentorship program for APSA trainees, who were paired with established physician-scientists for the opportunity for longitudinal mentor-mentee relationships. APSA is excited to continue working with ASCI and AAP toward developing innovative strategies to overcome new and existing barriers to successful physician-scientist training.

Only four years ago, APSA hosted the first physician-scientist trainee diversity summit. As we prepare to host a second summit with support from BWF, I want to take a moment to thank all the APSA members who have been vital in fulfilling the diversity goals established in June 2019: Jose Rodrigues, immediate past APSA president, oversaw the orchestration of many of these initiatives and played a critical role in launching the first iteration of VSRP; Bri Christophers, previous chair and current cochair of APSA’s JEDI committee who pioneered Mini Lessons, authored several manuscripts highlighting significant barriers to training and has led many of these initiatives in recent years; Briana Macedo, founder of VSRP, along with Eunice Lee and Michael Granovetter, founders of the Applicant Interactive Session series; Jennifer Kwan, for all the needs assessments she collected and published and associated advocacy across the physician-scientist pathway; this year’s Events committee chairs, Pearl Sutter and Zheng Hong Tan, who have ensured the successful launch of our partner initiatives and this Joint Meeting; my APSA team, Carey Jansen (vice-president), Alex Waldman (president-elect), and Amy Stark (executive director); and our board of directors, fantastic supporters of a large, ambitious, and trainee volunteer-led organization. APSA’s efforts in this arena are a work of passion, and I am grateful for the entire village.

So, “how do you fall into science?”

Like for many of my peers, falling into science wasn’t easy. The gravitational pull was enough to draw me in, but I don’t know where I would be without a few chance breaks. I recognize that there are many applicants into this career path for whom falling feels more like breaking into science. Ten years later, I’ve had the opportunity to lead APSA’s efforts toward change. We recognize that nothing but a multipronged approach to lessen this entry barrier will do. With Mini Lessons and a toolkit for local chapters, we increase early exposure to the physician-scientist pathway. With our mentorship programs and interactive sessions, we increase entry. With needs assessments, programming, and trainee mentorship, we hope to increase retention. In this way, our efforts to diversify the physician-scientist workforce persist; by helping people find the door and opening it a little wider, we’ll let gravity do its work.

## Figures and Tables

**Figure 1:**
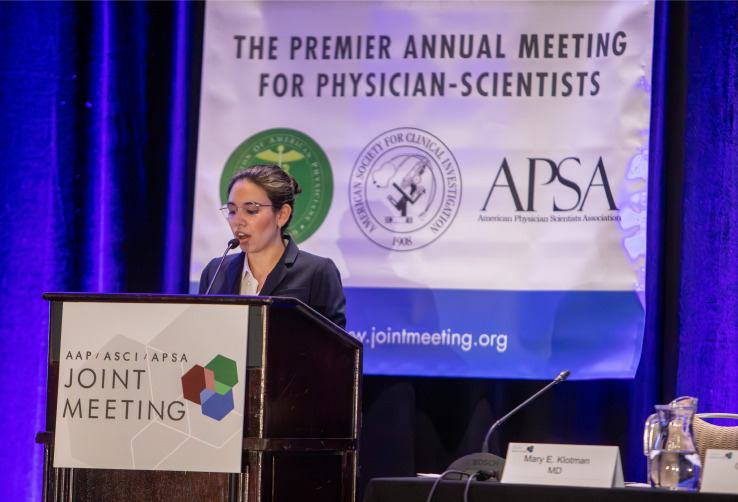
I delivered the American Physician Scientists Association (APSA) presidential address at the start of the AAP/ASCI/APSA Joint Meeting on April 21, 2023. *Bella Photography Studios*.

## Version 1 01/02/2024

Electronic publication
